# Molecular Characterization, Expression Profile, and A 21-bp Indel within the *ASB9* Gene and Its Associations with Chicken Production Traits

**DOI:** 10.3390/genes14020339

**Published:** 2023-01-28

**Authors:** Panpan Qin, Yang Liu, Xinran Niu, Yixuan Liu, Yushi Zhang, Yufang Niu, Yanxing Wang, Bingjie Chen, Ruili Han, Yadong Tian, Xiaojun Liu, Xiangtao Kang, Ruirui Jiang, Zhuanjian Li

**Affiliations:** 1 College of Animal Science and Technology, Henan Agricultural University, Zhengzhou 450001, China; 2 Key Laboratory of Livestock and Poultry Resources (Poultry) Evaluation and Utilization, Ministry of Agriculture and Rural Affairs, Zhengzhou 450001, China

**Keywords:** *ASB9*, chicken, growth and carcass trait, insertion/deletion, muscle

## Abstract

A growing number of studies have shown that members of the ankyrin repeat and suppressors of cytokine signaling (SOCS) box-containing protein (ASB) family are extensively involved in biological processes such as cell growth, tissue development, insulin signaling, ubiquitination, protein degradation, and skeletal muscle membrane protein formation, while the specific biological role of ankyrin-repeat and SOCS box protein 9 (*ASB9*) remains unclear. In this study, a 21 bp indel in the intron of *ASB9* was identified for the first time in 2641 individuals from 11 different breeds and an F_2_ resource population, and differences were observed among individuals with different genotypes (II, ID, and DD). An association study of a cross-designed F_2_ resource population revealed that the 21-bp indel was significantly related to growth and carcass traits. The significantly associated growth traits were body weight (BW) at 4, 6, 8, 10, and 12 weeks of age; sternal length (SL) at 4, 8, and 12 weeks of age; body slope length (BSL) at 4, 8, and 12 weeks of age; shank girth (SG) at 4 and 12 weeks of age; tibia length (TL) at 12 weeks of age; and pelvic width (PW) at 4 weeks of age (*p* < 0.05). This indel was also significantly correlated with carcass traits including semievisceration weight (SEW), evisceration weight (EW), claw weight (CLW), breast muscle weight (BMW), leg weight (LeW), leg muscle weight (LMW), claw rate (CLR), and shedding weight (ShW) (*p* < 0.05). In commercial broilers, the II genotype was the dominant genotype and underwent extensive selection. Interestingly, the *ASB9* gene was expressed at significantly higher levels in the leg muscles of Arbor Acres broilers than those of Lushi chickens, while the opposite was true for the breast muscles. In summary, the 21-bp indel in the *ASB9* gene significantly influenced the expression of the *ASB9* gene in muscle tissue and was associated with multiple growth and carcass traits in the F_2_ resource population. These findings suggested that the 21-bp indel within the *ASB9* gene could be used in marker-assisted selection breeding for traits related to chicken growth.

## 1. Introduction

With the advancement and rapid development of bioinformatics techniques, numerous genetic variants have been discovered in the genome, which can have different effects on individual traits, including single nucleotide polymorphisms (SNPs), insertions and deletions (indels), and copy number variations (CNVs). Currently, there is increasing interest in the relationship between mutations and growth traits, and researchers have conducted numerous studies on CNVs, indels, and SNPs in livestock and poultry genomes. A large number of mutant loci have been identified in animals, including cattle [1,2,3,4], sheep [5,6,7,8,9], pigs [10,11], and other livestock species, and these loci have become important markers for molecular breeding. Indel mutations have become increasingly important in recent years, and they have been widely applied in the genetic breeding of economic traits in livestock and poultry. Recent studies have identified many mutations related to growth and carcass traits in poultry [12,13,14,15]. At present, potential genes for Rhode Island Red chick feather color and stripe pattern have been revealed by a genome-wide association study (GWAS) [16]. GWAS can be used to identify indel polymorphisms of more functional genes for marker-assisted selection and to screen molecular markers associated with economic traits. Accurate information in molecular breeding can help increase the economic value of livestock and poultry and accelerate the genetic improvement of native chickens.

Numerous studies have demonstrated substantial connections between growth traits and quantitative trait loci (QTLs) at the end of chromosome 1 in various populations and breeds of chicken. Based on previous studies, we identified a 21-bp indel fragment at the end of GGA1 at 123,504,763 by resequencing and found that the mutant locus was located in the *ASB9* gene. We started to investigate the precise impacts of mutations at this locus on production traits and the expression of *ASB9* gene in different tissues.

Ankyrin repeat and SOCS box-containing 9 (*ASB9*) is a member of the ankyrin-repeat and suppressors of cytokine signaling (SOCS) box-containing (ASB) protein family (the largest family of SOCS box-containing proteins [17]). A total of 18 ASB family proteins, named ASB1-18, have been identified and are undergoing preliminary functional characterization. Members of the ASB family have two structural domains: an ankyrin protein tandem repeat (ANK) domain at the amino terminus, and a SOCS box domain at the carboxyl terminus [18]. Members of the ASB protein family can assist in protein degradation by forming ubiquitin ligase (E3) complexes with cullin and ring box proteins and by interacting with the elongin B-C bridging complex via its SOCS box structural domain [17,19]. It has been shown that members of this family are extensively involved in biological processes such as cell growth, tissue development, insulin signaling, ubiquitination and protein degradation, and skeletal muscle membrane protein formation [20,21,22,23]. *ASB9* has a common structure, including a unique N-terminus, an ankyrin protein repeat domain, and a SOCS box domain. Whereas the SOCS box interacts with Elongin B, Elongin C, and Cullin 5 in the E3 ligase complex, the ankyrin protein repeat domain and the N-terminal disordered fragment interact with creatine kinase [24] and are involved in processes such as ubiquitination and proteasomal degradation [25]. *ASB9* may serve as a biomarker for human breast cancer, in addition to its association with colorectal cancer [26,27]. According to a recent study, *ASB9* is primarily expressed in the kidney and testicles and plays a physiological role in bovine ovulatory follicles [25].

Although the functions of the ASB family have been extensively studied, the specific roles of ASB proteins in relation to the growth traits of chickens are not well understood, and the biological role of *ASB9* is still largely unknown. To date, it is unclear whether the role of *ASB9* in poultry growth and development is similar to that of other ASB family proteins. The purpose of this research was to determine how chicken growth and development are influenced by the gene polymorphism caused by the sixth intron 21-bp indel of the *ASB9* gene, as well as to create useful molecular genetic markers for broiler breeding. We examined the prevalence of this indel among 2641 individuals of 11 chicken breeds, as well as how the indel affected the growth and carcass characteristics of chickens in the F_2_ resource population. A reference for molecular marker-aided poultry breeding is provided by the *ASB9* gene, which can be utilized as a molecular marker in chickens.

## 2. Materials and Methods

### 2.1. Ethics Approval

All relevant international, national, and institutional regulations for the care and use of animals were adhered to. The Henan Agricultural University Institutional Animal Care and Use Committee (Permit Number: 11-0085) approved all procedures, which were carried out in accordance with the Chinese National Research Council Regulations from 1994.

### 2.2. Test Animals

#### 2.2.1. F_2_ Resource Population

The GS chickens and Anka (A) broilers were reciprocally crossed to produce an F_2_ resource population, where GS chickens represented slow-growing Chinese native chickens and Anka broilers represented fast-growing broilers. Forty-two grandparents were included in the F_2_ resource population (4 Anka 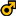 × 24 GS chickens 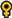, forward cross; 2 GS chickens 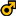 × 12 Anka 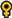, backward cross; and 70 F_1_ individuals were obtained). To improve the segregation of F_2_ characteristics, animals with outstanding phenotypes and strong heterozygosity were chosen from this cross combination, and up to nine hens, chosen at random, were mated with each rooster in a 1:9 male to female ratio.

In total, 795 F_2_ chickens from seven families, were produced (including 491 individuals from four orthologous families with Anka chickens as the male parent, and 304 individuals from three reverse cross families with GS chickens as the male parent). All chickens were provided feed and water ad libitum under the same conditions and slaughtered on reaching 12 weeks of age. From hatching to 12 weeks of age (slaughter age), the growth and carcass traits [28] were monitored and documented in the F_2_ individuals. These traits included body weight (BW) at ages 0, 2, 4, 6, 8, and 12 weeks; tibia length (TL) at ages 0, 4, 8, and 12 weeks; and shank girth (SG), chest width (ChW), chest depth (CD), sternum length (SL), body slope length (BSL), and pelvic width (PW) at ages 4, 8, and 12 weeks. After slaughter, carcass traits and growth traits were measured and collected as follows: breast muscle weight (BMW), leg weight (LeW), shedding weight (ShW), leg muscle breadth (LMB), leg muscle density (LMD), leg muscle weight (LMW), semievisceration weight (SEW), evisceration weight (EW), sebaceous weight (SeW), head weight (HW), claw weight (CLW), double wing weight (DWW), liver weight (LW), heart weight (HeW), gizzard weight (GW), spleen weight (SW), pancreas weight (PW), and carcass weight (CW) [29,30]. Moreover, blood samples from the F_2_ resource population were obtained at 12 weeks of age and kept at −80 °C until further examination. Alanine aminotransferase (ALT) was measured via colorimetric enzymatic methods. Details of the cross construction, feeding management, determination of serum variables, and slaughter of the F_2_ population can be found in previous studies [15].

#### 2.2.2. Genotyping Sample Collection

A total of 2641 blood samples were taken for our study, as shown below: the F_2_ resource population (*n* = 654), dual-purpose chickens (Dongxiang chickens (DX, *n* = 189), Changshun chickens (CS, *n* = 181), Lushi chickens (LS, *n* = 271), Yunyang chickens (YY, *n* = 77), Guifei chickens (GF, *n* = 264), Henan gamecock (HNG, *n* = 84)), commercial broilers (Ross 308 broilers (RS308, *n* = 92), HBD broilers (HBD, *n* = 96), Arbor Acres broilers (AA, *n* = 279) and Cobb broilers (Cobb, *n* = 182)), and commercial laying hens (Hy-Line variety brown (HL, *n* = 272)). Blood samples from these different individuals were provided by the Key Laboratory of Livestock and Poultry Resources (Poultry) Evaluation and Utilization of Henan Agricultural University. The blood samples were taken for DNA extraction, with subsequent genotyping.

In addition, samples from chickens at diverse stages of development were gathered for RNA analysis, to verify the transcript expression pattern of *ASB9* in the various breeds: tissue samples from embryonic day-14 (E14) AA broilers (commercial broilers) (heart, liver, spleen, breast muscle, leg muscle, pancreas, and muscle stomach), tissue samples from 3-week-old AA broilers and LS (local Chinese chickens) (heart, liver, spleen, lung, kidney, breast muscle, leg muscle, pancreas, and muscle stomach), samples from AA broilers and LS at different developmental stages (embryonic days 10, 12, 14, 16, and 18, as well as 1-day-old, 1-week-old, 3-week-old, and 5-week-old), and leg muscle samples (embryonic days 10, 12, 14, 16, and 18, as well as 1-day-old, 1-week-old, and 3-week-old) and liver samples (1-day-old, 1-week-old, 3-week-old and 5-week-old) from AA broiler chickens and LS at different developmental stages. Table 1 lists the samples. For the purpose of determining the relative gene expression levels of *ASB9* for the various breeds, tissues, and periods of time, RNA was isolated from cells and tissues. RNA and all tissues were kept in a refrigerator at −80 °C.

#### 2.2.3. Cell Culture

As previously mentioned, we also isolated chicken primary myoblasts from the leg muscle of 11-day-old AA broiler embryos [31]. The cells were grown in high-glucose DMEM (Biological Industries, Beit Haemek, Israel) containing 15% FBS and 1% penicillin/streptomycin (Solarbio, Beijing, China) until the second generation of cells had reached >90% confluence and then changed to differentiation medium (DMEM containing 2% horse serum) for further culture. An inverted microscope was used to observe the cell status. Cells were maintained at 37 °C in a 5% CO_2_ environment, and cell samples were collected at 1, 2, 3, 4, 5, 6, and 7 days of differentiation induction.

### 2.3. DNA Isolation and PCR

Fresh whole blood samples were collected in EDTA-K_2_ vacutainer tubes, and genomic DNA was isolated using the phenol-chloroform procedure [32]. Using gel electrophoresis and a NanoDrop 2000 spectrophotometer (Thermo, Waltham, MA, USA), the quality and quantity of total DNA were determined. The DNA samples were frozen at −20 °C after being diluted to a concentration of roughly 10 ng/L.

The NCBI online primer design tool (http://www.ncbi.nlm.nih.gov/tools/primer-blast/, accessed on 6 July 2021) was used to design primers for PCR and real-time quantitative polymerase chain reaction PCR (qRT-PCR) analysis, and they were synthesized by Sangon Biotech Company (Sangon, Shanghai, China). Table 2 provided information on all primer sequences, projected product lengths, and Tm values. The internal benchmark for normalization was *GAPDH*. Different breeds of chickens were genotyped using the *ASB9*-F1/R1 primers. In the *ASB9* gene’s intron, these primers produced a 60-bp/81-bp DNA fragment. The expression levels of the *ASB9* gene in each tissue were assessed using *ASB9*-qF1/qR1 primers. A 10 μL volume with 50 ng of DNA, 5 μL of 2×Taq Master Mix (Kangwei, Beijing, China), 0.5 μL of each primer, and 3 μL of dd water were used to perform the PCR. PCR amplification was carried out using the following profile: 95 °C for 5 min; then 95 °C for 30 s, 60 °C for 30 s, 72 °C for 30 s (35 cycles); 72 °C for 10 min; and a 4 °C holding temperature.

### 2.4. RNA Extraction, cDNA Synthesis, and qPCR

qPCR was used to examine the *ASB9* gene’s mRNA expression in diverse tissues. Using the TRIzol^®^ reagent (Takara, Kyoto, Japan), total RNA was isolated from the aforementioned samples. The RNA concentration and purity were determined using the 260 nm/280 nm optical density (OD) absorption ratio using a NanoDrop 2000c spectrophotometer (Thermo Fisher Scientific, Waltham, MA, USA), and the samples with a ratio control in the range 1.9 to 2.0 were chosen for cDNA synthesis. cDNA was prepared with HiScript III RT SuperMix for qPCR (+gDNA wiper) (R323–01, Vazyme Biotech Co., Ltd., Nanjing, China). SYBR Green qPCR Mix (Q711, Vazyme Biotech Co., Ltd., Nanjing, China) was used in a two-step procedure to carry out PCR using an ABI 7500 instrument (Applied Biosystems, Foster City, CA, USA), in accordance with the manufacturer’s recommendations. Using a Roche LightCycler^®^ 96 (Roche, Basel, Switzerland) instrument, qPCR was carried out in each 10 μL reaction using 0.4 μL of a 10 μM primer mix and 25 ng of cDNA, under the following conditions: 95 °C for 5 min, followed by 35 cycles of 95 °C for 20 s, 60 °C for 30 s, and 72 °C for 30 s. GraphPad Prism 8.0.1 (GraphPad Software Inc. 2018, San Diego, CA, USA) was used to draw the figures. The 2^−ΔΔCt^ method and one-way ANOVA followed by Duncan’s test were used to examine the relative expression of gene and significant differences in gene expression among the various developmental stages and tissues, respectively [33,34].

### 2.5. Statistical Analysis

On the basis of the selected indel variants, raw genotypes and allele frequencies were calculated in eleven chicken breeds and the F_2_ resource population. Nei’s method was used to compute genetic diversity indexes (homozygosity, Ho; heterozygosity, He; (Ho + He = 1); polymorphism information content, PIC) [35]. The Bonferroni test was used for multiple comparisons [36,37]. The SHEsis website (http://analysis.bioxbiox.cn, accessed on 22 June 2021) was used to calculate the Hardy–Weinberg equilibrium (HWE) [38]. Analysis of the DNA sequences was performed using BioXM (version 2.7) and DNAMAN (version 7.0).

According to two linear mixed models, as we previously reported, the relationships of diverse genotypes with production traits in the F_2_ generation population were examined using SPSS software (SPSS for Windows, standard version 25.0; Chicago, IL, USA). Model I was used to evaluate the growth traits, meat quality traits, and serum biochemical indicators. Model II was used to analyze carcass traits, with carcass weight (CW) as a control variable, in order to take into account how body weight affected the carcass traits.

Model I: Y_ijklm_ = μ + G_i_ + S_j_ + H_k_ + f_l_ + e_ijklm_

Model II: Y_ijklm_ = μ + G_i_ + S_j_ + H_k_ + f_l_ + b (W_ijklm_ − W (—)) + e_ijklm_

These models use Y_ijklm_ to represent the observed values, µ to represent the population’s overall mean, and G_i_ to represent the fixed genotype impact (I = 3), which includes both additive and dominant gene effects (for the II, ID, and DD genotypes, the additive effect values were −1, 0, and 1, and the dominating effect values were 1, −1, and 1, respectively). The fixed effect of sex is S_j_ (j = 2), and the fixed effect of the hatch is H_k_ (k = 1, 2). W_ijklm_ stands for individual slaughter weight, W (—) for mean slaughter weight, and e_ijklm_ for random error. f_l_ is the fixed effect of family, b is the regression coefficient for carcass weight. All data in our study are reported as the SEM ± mean, *p* < 0.05 was deemed statistically significant, and least squares analysis was used to examine the impact of polymorphism genotypes on the target traits.

### 2.6. Phylogenetic Analysis

To investigate the conservation of the *ASB9* gene across species, the amino acid sequences of the *ASB9* gene from the GRCg7b version of the chicken genome (*Gallus gallus*) and its homologs in 25 species, including tropical clawed frog (*Xenopus tropicalis*), wild yak (*Bos mutus*), human (*Homo sapiens*), house mouse (*Mus musculus*), and mallard (*Anas platyrhynchos*) were downloaded from the NCBI website. Then, ClustalX2.1 was used to conduct multiple sequence alignments of the *ASB9* gene proteins from various species (Table S1, Supplementary Materials), and MEGA 7.0 was used to create phylogenetic trees with 1000 bootstrap repetitions. Finally, an evolutionary tree was constructed using EvolView (https://evolgenius.info//evolview-v2/#login, accessed on 20 September 2022).

## 3. Results

### 3.1. Molecular Characterization and Bioinformatics Analysis of ASB9 in Chickens

**Figure 1:**
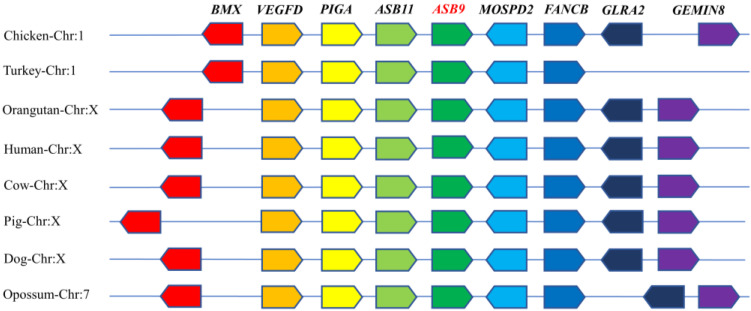
Synteny analysis of *ASB9* genes from various species. Genes are represented by various colors.

First, we investigated the location of the *ASB9* gene. The chicken *ASB9* gene is found on chromosome 1 (GenBank accession number NC_052532.1) and consists of two variable spliceosomes that contain eight exons and encode a protein with 294 amino acids. The gene contexts in twelve different species were examined using synteny analysis (Figure 1). The findings revealed that the genomic area surrounding the *ASB9* gene is conserved among species and includes numerous genes with high levels of conservation, such as *BMX* (ENSGALG00000016557), *VEGFD* (ENSGALG00000016558), *PIGA* (ENSGALG00000016559), *ASB11* (ENSGALG00000016562), *MOSPD2* (ENSMGAG00000016568), *FANCB* (ENSMGAG00000016569), *GLRA2* (ENSGALG00000016571), and *GEMIN8* (ENSGALG00000016574), which suggests that *ASB9* is an important gene.

**Figure 2:**
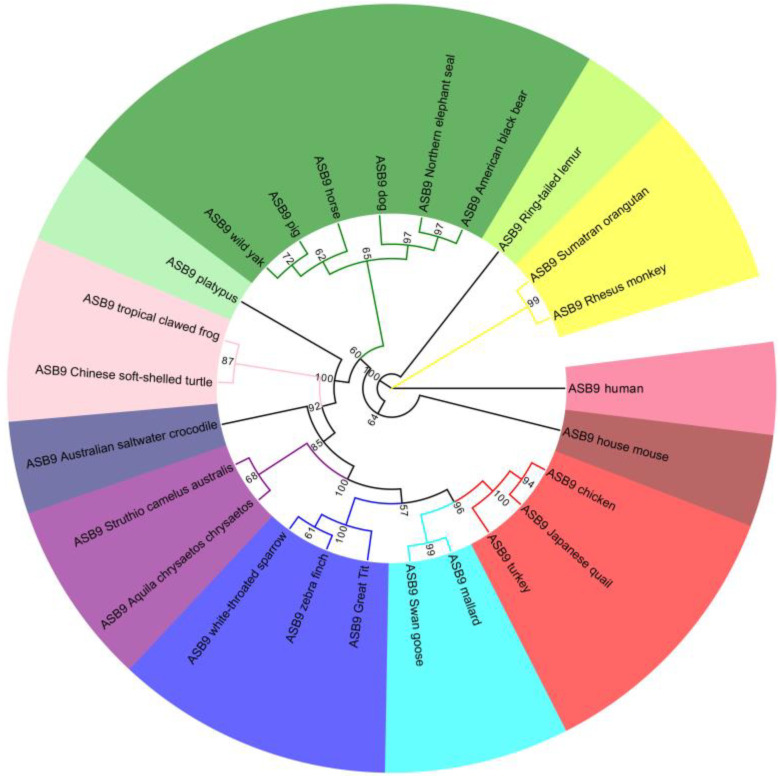
Phylogenetic analysis of the *ASB9* gene. A maximum likelihood tree (ML) was created using ClustalX2.1 and MEGA 7.0 and visualized with EvolView.

Phylogenetic trees were constructed using MEGA software and the maximum likelihood (ML) method, in order to further study the evolutionary relationships of *ASB9* genes among the various species (Figure 2). We analyzed the amino acid sequences of *ASB9* in 25 species, including tropical clawed frog (*X. tropicalis*), wild yak (*B. mutus*), human (*H. sapiens*), house mouse (*M. musculus*), and mallard (*A. platyrhynchos*). The phylogenetic trees showed that the *ASB9* gene of chickens was evolutionarily conserved and was most closely related to those of Japanese quail (*Coturnix japonica*) and turkey (*Meleagris gallopavo*), and more distantly related to those of human, house mouse, ring-tailed lemur, Sumatran orangutan, and rhesus macaque, consistent with the overall evolutionary distances among these species.

### 3.2. Identification of Genetic Variants Correlated with ASB9 Expression

**Figure 3:**
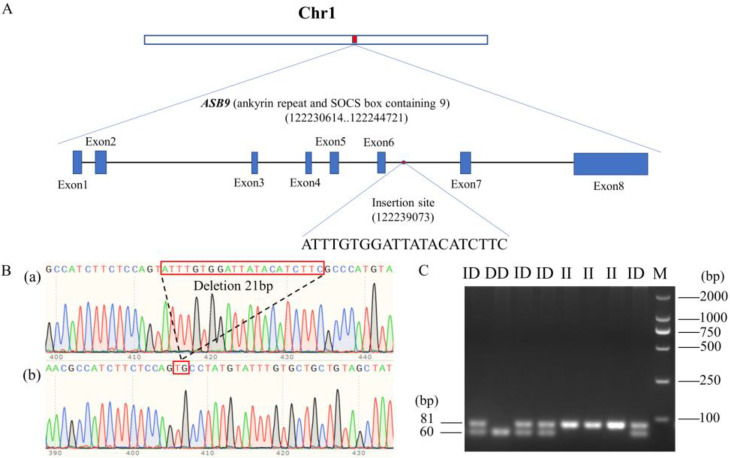
Identification of genetic variants associated with *ASB9* expression. (**A**) The insertion site and relative location of the chicken *ASB9* gene. The insertion site is situated in the sixth intron of the *ASB9* gene, which is situated in the middle of chicken chromosome 1. (**B**) Sequence diagrams for the *ASB9* gene’s 21-bp indel loci. The sequence diagrams for the I and D alleles are shown in (a,b), respectively. (**C**) Agarose gel electrophoresis revealed genotyping patterns for the *ASB9* 21-bp indel polymorphism. M refers to marker; II to insertion type homozygote; ID to heterozygous type; and DD to deletion type homozygote.

A novel 21-bp insertion/deletion (GenBank accession number NC_052532.1, 122239073-122239093) mutation was detected in the *ASB9* gene using whole-genome resequencing (Figure 3A). We sequenced the *ASB9* gene’s sixth intron region in Gushi (GS) chickens where the mutation occurred. A description of the mutant sequence was given as NC_052532.1 ins 122239072-122239073 ATTTGTGGATTATACATCTTC, as shown in Figure 3B. After amplification of the PCR results, a 2.0% agarose gel electrophoresis was used to detect the indel polymorphisms. The PCR results revealed all three genotypes, which were II (81 bp), ID (81 bp and 60 bp), and DD (60 bp), as shown in Figure 3C. A detailed description of the PCR primers used to amplify the indel locus is presented in Table 2.

### 3.3. The ASB9 Gene 21-bp Indel Locus Genotypes and Genetic Parameters in Eleven Chicken Breeds and an F_2_ Resource Population

A novel 21-bp insertion sequence was discovered in the chicken *ASB9* gene, according to electrophoretic gel mapping. Therefore, it was important to examine the relationships between the growth traits and genotype at the 21-bp indel locus in a larger chicken population. In 2641 individuals, the *ASB9* indel locus’s genetic parameters (allele frequencies, observed heterozygosity, Hardy–Weinberg equilibrium results, effective allele numbers, and polymorphism information content) were calculated. The F_2_ resource populations, commercial broilers (Ross 308, HBD, Cobb, and AA), dual-purpose chickens (DX, CS, LS, YY, GF, and HNG), and commercial laying hens (HL) were all included in this analysis.

Diversity at the genetic level is usually described by the number and frequency of alleles per locus in a population. As compared to allele D, which had a lower frequency (F_2_/28.4%, Ross 308/34.2%, HBD/38.5%, AA/38.8%, DX/46.0%, CS/45.6%, LS/35.2%, YY/48.1%, HL/47.6%), allele I (F_2_/71.6%, Ross 308/65.8%, HBD/61.5%, AA/61.2%, DX/54.0%, CS/54.4%, LS/64.8%, YY/51.9%, HL/52.4%) was more common, except in the GF, HNG, and Cobb populations. In comparison to commercial laying hens (HL/19.0%) and dual-purpose chickens (DX/29.0%, CS/27.0%, YY/25.0%, and HNG/11.0%), the Ross 308 (41.0%) and HBD (31.0%) breeds had higher frequencies of the II genotype. As expected, the frequency of the DD genotype (GF/31%, HNG/24%) was higher than that of the II genotype (GF/19%, HNG/11%) (Table 3). These findings revealed that genotype II, which has been selected for in commercial broilers and dual-purpose chickens, was the dominant genotype. Although, commercial laying hens still have a lot of room for breeding. A hybridization method can greatly increase the growth performance of dual-purpose chickens, as evidenced by the fact that the frequency of the I allele was higher in the F_2_ resource population than in the dual-purpose chickens.

The HWE for this locus included the DX, CS, LS, YY, GF, and Ross 308 chickens (*p* > 0.05). Conventionally, a PIC > 0.5 denotes high polymorphism, a PIC between 0.25 and 0.5 denotes intermediate polymorphism, and a PIC below 0.25 denotes low polymorphism. PIC and Ho are measures of genetic diversity within the population, as well as the degree of allelic polymorphism and gene mutation. Only intermediate polymorphism (0.25 ≤ PIC ≤ 0.5) was found at the mutant loci of the *ASB9* gene in all breeds of chicken analyzed, indicating that these breeds had more genetic diversity and selection potential.

### 3.4. Association of the ASB9 Gene 21-bp Indel with Growth, Carcass, and Blood Biochemical Variables

**Figure 4:**
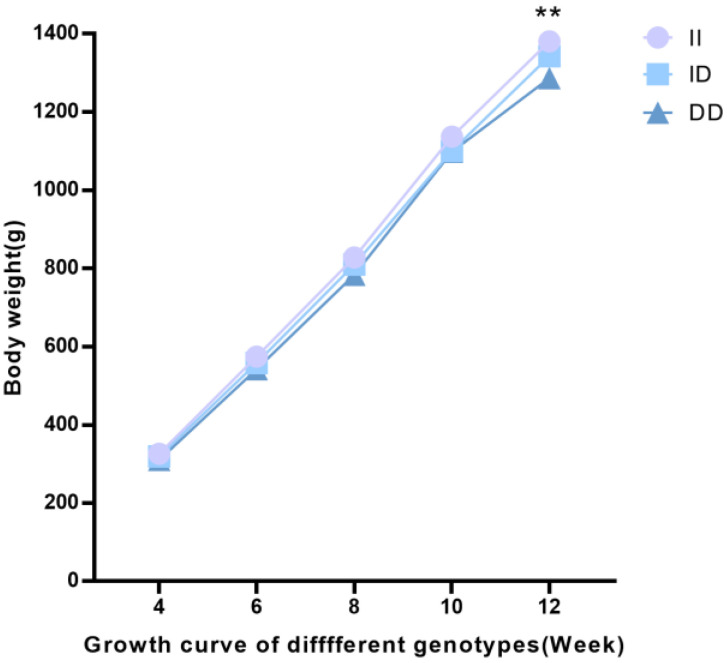
Body weight varies over time for various *ASB9* genotypes in the F_2_ resource population at different weeks. ** *p* < 0.01.

According to the results of the correlation analysis, the 21-bp indel polymorphism was substantially related to a variety of carcass traits and growth traits in the F_2_ resource population. This indel had a significant impact on growth traits at the following ages: BW at 4, 6, 8, and 10 weeks of age; BSL at 4, 8, and 12 weeks of age; PW at 4 weeks of age; TL at 12 weeks; SG at 4 and 12 weeks; and SL at 4 and 8 weeks of age (*p* < 0.05). A highly significant connection was also found between 12-week BW and SL (*p* < 0.01; Table 4). Interestingly, from 4 to 12 weeks of age, II genotype individuals weighed more than ID and DD genotype individuals (Figure 4). There were no discernible differences found between individuals with the ID genotype and those with the DD genotype.

This indel significantly affected the carcass traits SEW, EW, BMW, HWP, CLR, ShW, LMB, and LMD (*p* < 0.05), as well as CLW, LeW, LMW, and PWP (*p* < 0.01; Table 5). The carcass traits also differed among genotypes; while ID genotype individuals were statistically indistinguishable from DD genotype individuals, the II genotype chickens had higher SEW, EW, BMW, CLW, CLR, LeW, LMW, LMB, and ShW values than the ID and DD genotype chickens.

The indel was substantially correlated to alanine aminotransferase (ALT) according to the findings of the serum variable association study (*p* < 0.01; Table 6). These findings indicated that the 21-bp indel significantly affected chicken growth and development.

### 3.5. Expression Level of ASB9 in Chickens

**Figure 5:**
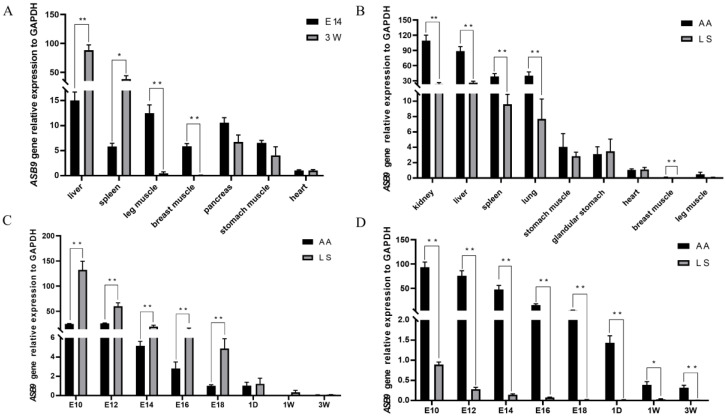
*ASB9* gene expression relative to *GAPDH*. (**A**) Relative gene expression levels of *ASB9* in E14 and 3-week-old tissues from AA broilers. *n* = 6. (**B**) Relative gene expression levels of *ASB9* in 3-week-old tissues from LS and AA broilers. *n* = 6. (**C**) Relative gene expression levels of *ASB9* at E10, E12, E14, E16, E18, 1 day, 1 week, and 3 weeks in the breast muscle tissues of LS and AA broilers. *n* = 6. (**D**) Relative gene expression levels of *ASB9* at E10, E12, E14, E16, E18, 1 day, 1 week, and 3 weeks in the leg muscle tissues of LS and AA broilers. *n* = 6. LS: Lushi chickens; AA: Arbor Acres broilers; * *p* < 0.05, ** *p* < 0.01.

Since *ASB9* was highly correlated with various growth traits, it was hypothesized that *ASB9* might be connected to the growth traits of chickens. According to the genotyping results, LS was selected as a representative breed of dual-purpose chickens and the AA broiler as a representative breed of commercial broilers, to verify the expression of *ASB9* in different breeds. Three-week-old LS chickens and E14 and AA commercial broilers tissues were both examined for *ASB9* mRNA expression. Notably, throughout the embryonic stage, the liver, leg muscles, pancreas, and breast muscles all showed higher levels of *ASB9* gene expression (Figure 5A). We found the same expression patterns in AA broilers and LS chickens at 3 weeks of age, with lower expression levels in the breast muscles and leg muscles and higher expression levels in the kidney and liver (Figure 5B). Additionally, except for the muscle stomach and heart, the *ASB9* gene expression levels in AA broilers were considerably higher than those of LS chickens, suggesting a potential relationship between *ASB9* expression levels and growth rate.

In AA broilers and LS chickens, the expression of the *ASB9* gene was investigated at the following developmental stages: E10, E12, E14, E16, E18, 1 day, 1 week, and 3 weeks (Figure 5C,D). *ASB9* expression in breast muscles and leg muscles gradually decreased in successive developmental stages. In addition, the expression of *ASB9* was significantly higher in the breast muscle tissues of LS chickens than in AA broilers throughout the embryonic period. Interestingly, the opposite result was observed in the leg muscle, where the expression level of the *ASB9* gene was significantly higher in the leg muscle of AA broilers than in LS chickens from E10 to 3 weeks.

**Figure 6:**
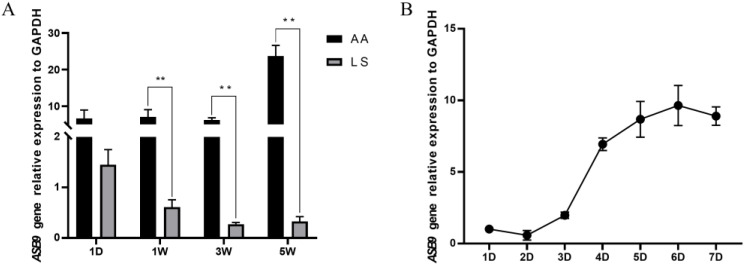
*ASB9* gene expression relative to *GAPDH*. (**A**) Relative gene expression levels of *ASB9* at 1 day, 1 week, 3 weeks, and 5 weeks in the liver tissues of LS and AA broilers. LS: Lushi chickens; AA: Arbor Acres broilers. *n* = 6. (**B**) Relative expression level of the *ASB9* gene during myoblast differentiation in AA broilers; AA: Arbor Acres broilers. *n* = 6; ** *p* < 0.01.

Since the expression of *ASB9* was relatively high in the liver tissues of AA broilers and LS chickens, we examined the expression of the *ASB9* gene in the liver tissues of AA broilers and LS chickens at 1 day, 1 week, 3 weeks, and 5 weeks (Figure 6A). We discovered that the liver tissues of AA broilers and LS chickens showed opposing patterns of *ASB9* expression. The expression of *ASB9* in the liver tissues of LS chickens gradually decreased with increasing age, while in AA broilers, no obvious differences were observed between 1 day, 1 week, and 3 weeks, and the highest expression was observed at 5 weeks.

Furthermore, since the embryonic leg muscle displayed higher *ASB9* gene expression levels, we isolated and cultured chicken primary myoblasts from the AA broiler leg muscle of E10 embryos, to construct an induced chicken primary myoblast differentiation model, and examined the change in the levels of *ASB9* mRNA expression at 1, 2, 3, 4, 5, 6, and 7 days after cell differentiation (Figure 6B). The expression of *ASB9* in chicken primary myoblasts tended to increase with the growth cycle. Specifically, the expression level was lowest at 2 days and increased rapidly with further development, reaching a peak at 6 days, and then tending to decrease over the later stages of differentiation. These results indicated that the *ASB9* gene might influence muscle development by promoting the differentiation of chicken myoblasts, further indicating that *ASB9* may have an impact on chicken development and growth.

## 4. Discussion

In recent years, the effects of random mutations within introns on gene function have been increasingly recognized, and the functions of gene sequences have been studied in depth [39,40]. Studies have also revealed that introns contain a variety of non-coding RNAs, in addition to elements linked to gene transcription and regulation, such as alternative splicing and mRNA processing [41,42]. Numerous studies have demonstrated that intron mutations are related to disease phenotypes, growth, and reproduction in livestock and poultry [43,44]. *IGF2* (insulin-like growth factor 2) gene mutations in the intron are significantly linked to the accumulation of fat and muscle [45]. Studies in chickens have also shown that mutations in the introns of the *PAX7*, *KLF15*, and *YBX3* genes are correlated with chicken carcass and growth characteristics [12,14,46]. Furthermore, recessive white mutations in chickens are associated with retroviral insertion in the tyrosinase gene’s intron 4 [47]. In this study, a novel 21 bp indel mutation in the *ASB9* gene’s intron region was discovered to be strongly associated with growth traits, slaughter traits, and serum variables in a cross-designed F_2_ resource population. These findings show the significance of this indel for the growth and development of poultry, particularly for body weight.

Growth traits, which are the manifestation of intricate biological changes in the organism, are impacted by many different variables, such as genetics, nutrient uptake, feeding practices, etc. [48]. Artificial breeding primarily focuses on traits related to the chicken growth and carcass, which are economically significant in the production of poultry. In the production of broilers, the breast muscle weight (BMW) and leg weight (LeW) are crucial factors, and higher phenotypic values can have a positive impact on the economic outcome. GWASs have found several new genes and genetic variation sites related to breast muscle development [49], as well as the genetic architecture and important variants for breast muscle weight in native chickens [50]. According to the findings of this study, *ASB9* gene polymorphisms in the cross-designed F_2_ population were significantly associated with SEW, EW, BMW, HWP, CLR, ShW, LMB, and LMD (*p* < 0.05), as well as CLW, LeW, LMW, and PWP (*p* < 0.01). Body weight is a crucial quantitative trait with a high heredity, reflects animal growth and development, and has a direct impact on nutrition absorption, fat and protein deposition, and bone development and growth [51]. The results of this study suggest that mutation at this locus was significantly associated with body weight at 4, 6, 8, 10 (*p* < 0.05), and 12 (*p* < 0.01) weeks of age. From 4 to 12 weeks, II genotype individuals had an average body weight that was higher than the DD genotype individuals. At 12 weeks of age, II genotype individuals had an average body weight that was relatively high than the DD genotype and ID genotype individuals. This finding indicated that the reciprocal cross F_2_ population was significantly dominated by the II genotype.

Many economic traits in poultry are quantitative traits. The occurrence of such traits is controlled by two or more pairs of alleles, and the genetic basis of quantitative traits is a minor polygenic system [52]. Growth traits are extremely complex quantitative traits in poultry. There is no significant difference in the II genotype frequency between broiler and F_2_ resource populations, suggesting that the variation in phenotypic values for these quantitative traits may be controlled by minor polygenes, with the I allele playing a minor role in the expression of these quantitative traits and influenced by other genetic factors. The II genotype was significantly more common in broiler breeds compared to commercial laying hens and dual-purpose chickens, except in Cobb broilers and LS chickens, where the ID genotype frequencies were higher than in local chickens, probably due to insufficient selection intensity. Overall, the I allele was selected for commercial broilers. It is noteworthy that the II genotype frequency was lower than the DD genotype frequency in GF chickens and Henan gamecocks. GF chickens are small and high quality, with thin skin, fine bones, and little fat, while purebred Henan gamecocks have a small body size and poor physical endurance, and neither GF chickens nor Henan gamecocks are selected for size during breeding. Although the data showed selection for the I allele in commercial broilers, the polymorphism rates (0.25 ≤ PIC ≤ 0.5) were moderate in all populations, indicating that this indel locus still has great potential for selection in different populations.

The specific mechanism through which *ASB9* affects biological growth and development is not clear. The active protein genes containing the SOCS box protein domain in muscle differentiation have attracted much attention from researchers. It has also been shown that SOCS box-containing domains can regulate the phosphorylation of growth hormone receptor (GHR), Janus kinase (JAK), and signal transducer and activator of transcription (STAT) proteins; can affect growth hormone (GH) signaling pathway responses; and are crucial for the regulation of animal growth and development, as well as muscle differentiation [53,54]. In addition, it has been reported that the ankyrin (ANK) repeat domain has an inhibitory effect on myogenesis [55]. Recent reports show that *ASB9* regulates ovarian granulosa cell function and mitogen-activated protein kinase (MAPK) signaling [56]. *ASB9* interacts with a creatine kinase (CK) dimer in an asymmetric manner [57,58]. In human embryonic kidney 293 (HEK293) cells, the interaction of *ASB9* with the CK system induced mitochondrial malfunction and was shown to negatively regulate cell growth [59]. CK is an important cytoplasmic enzyme in cellular energy metabolism and is evolutionarily conserved [60]. CK is associated with brain and muscle function and reversibly catalyzes the adenosine triphosphate (ATP) phosphorylation of creatine, thereby regulating ATP levels in tissues that require large amounts of energy and playing an essential role in the cellular homeostasis of the vertebrate heart and brain [61,62,63]. This interaction between CK and *ASB9* was confirmed to be specific to *ASB9*, suggesting that *ASB9* may be involved in cellular energy metabolism and muscle development. In our study, *ASB9* expression was relatively high in the liver and muscle, suggesting that *ASB9* may play a similar role in chickens.

The study of gene expression levels is an important component of determining markers for molecular breeding [64]. To further explore the reasons for the observed phenotypic associations with the 21-bp deletion, we contrasted how *ASB9* mRNA was expressed in various tissues from various species at various times. The findings demonstrated that the *ASB9* expression varied, being higher in the liver, breast muscles, and leg muscles during the embryonic stages and lower in the heart and stomach muscles. Based on these findings, we speculated that *ASB9* expression might affect muscle development positively.

Notably, the expression of *ASB9* in breast muscles and leg muscles was significantly lower in chicks at 3 weeks of age, and *ASB9* seemed to function at different times in different tissues. Meanwhile, we found that the tissue expression patterns of *ASB9* in AA broilers and LS chickens were the same, although the expression levels of the *ASB9* gene were significantly higher in AA broilers than in LS chickens, except in the muscle, stomach and heart tissues. Additionally, *ASB9* expression gradually increased with development in the liver tissues from AA broilers, while the expression in the liver tissues of LS chickens showed opposite trends at different periods. LS is a local breed in Henan Province, with slow growth and high-quality meat, while the AA broiler is a representative fast-growing broiler. Both meat quality and growth rate differ greatly between local chickens and commercial broilers. It was speculated that the 21-bp indel in *ASB9* might promote broiler growth and development through effects on energy metabolism. According to our research, *ASB9* appears to play a role in the early stages of chicken embryo development and functions mainly from 0 to 5 weeks after birth.

Overall, our findings indicate that the *ASB9* gene may have a profound effect on production traits, but further studies are necessary, to verify allelic effects in further populations.

## 5. Conclusions

In summary, the present study strongly supports that the 21-bp indel in the *ASB9* gene is significantly associated with several growth and carcass traits in chickens. Genotype II may be a dominant genotype and is significantly and positively correlated with growth and carcass traits. *ASB9* mRNA expression is enriched in the embryonic liver, pectoral muscle, and leg muscle, and this suggests that *ASB9* may participate in embryonic development by promoting muscle growth. These findings contribute to a deeper understanding of the relationship between *ASB9* polymorphisms and poultry economic traits. Further research using bioinformatics to validate these conclusions in hybrid populations may be considered.

## Figures and Tables

**Table 1:** Samples of tissue and cells used for RNA extraction.

Breeds	Tissue/Cell Samples	Week Age
AA	heart, liver, spleen, breast muscle, leg muscle, pancreas, muscle stomach	E14
AA	heart, liver, spleen, lung, pancreas, muscle stomach, breast muscle, leg muscle, and kidney	3W
LS	heart, liver, spleen, lung, pancreas, muscle stomach, breast muscle, leg muscle, and kidney	3W
AA	breast muscle	E10, E12, E14, E16, E18, and 1D, 1W, 3W
AA	leg muscle	E10, E12, E14, E16, E18, and 1D, 1W, 3W
LS	breast muscle	E10, E12, E14, E16, E18, and 1D, 1W, 3W
LS	leg muscle	E10, E12, E14, E16, E18, and 1D, 1W, 3W
LS	liver	1D, 1W, 3W, 5W
AA	primary myoblasts	1D, 2D, 3D, 4D, 5D, 6D, 7D

Arbor Acres broilers (AA); Lushi chicken (LS); E10, E12, E14, E16, E18, and 1D, 2D, 3D, 4D, 5D, 6D, 7D, 1W, 3W, 5W (embryonic ages of 10, 12, 14, 16, 18 days and 1 day, 2 days, 3 days, 4 days, 5 days, 6 days, 7 days, 1 week, 3 weeks, and 5 weeks of age).

**Table 2:** PCR primers for amplifying the *ASB9* gene in chickens.

Primer Set	Primer Sequence, (All 5′-3′)	Product Size (bp)	Tm, °C
*ASB9*-F1	TGCCTAGAACAACGCCATCTT	60/81	60
*ASB9*-R1	TCAAATAGCTACAGCAGCACAA		
*ASB9*-qF1	TGAATTTACTGCTGCAACATGGG	293	60
*ASB9*-qR1	CACCAGCTCTACACTGCAAC		
*GAPDH*-F	GAACATCATCCCAGCGTCCA	132	60
*GAPDH*-R	CGGCAGGTCAGGTCAACAAC		

**Table 3:** Genetic parameters of 21 bp locus loci within the *ASB9* gene in the F_2_ and eleven other chicken breeds.

Breeds		*n*	Genotype and Allelic Frequencies					Ho	Ne	PIC	*p*-Value, HWE
			II	ID	DD	I	D				
F_2_ generation resource population	F_2_	654	0.49	0.46	0.05	0.716	0.284	0.460	1.685	0.32	0.00
Dual-purpose chickens	DX	189	0.29	0.50	0.21	0.540	0.460	0.497	1.987	0.37	0.99
	CS	181	0.27	0.55	0.18	0.544	0.456	0.547	1.984	0.37	0.17
	LS	271	0.41	0.48	0.11	0.648	0.352	0.476	1.840	0.35	0.48
	YY	77	0.25	0.55	0.21	0.519	0.481	0.545	1.997	0.37	0.42
	GF	264	0.19	0.50	0.31	0.438	0.563	0.504	1.969	0.37	0.70
	HNG	84	0.11	0.65	0.24	0.435	0.565	0.655	1.966	0.37	0.00
Commercial broilers	RS308	92	0.41	0.49	0.10	0.658	0.342	0.489	1.819	0.35	0.41
	HBD	96	0.31	0.60	0.08	0.615	0.385	0.604	1.900	0.36	0.01
	AA	279	0.26	0.70	0.04	0.612	0.388	0.702	1.904	0.36	0.00
	Cobb	182	0.15	0.69	0.16	0.492	0.508	0.687	1.999	0.37	0.00
Commercial laying hens	HL	272	0.19	0.66	0.15	0.524	0.476	0.658	1.995	0.37	0.00

F_2_ generation resource population (F_2_), Dongxiang chicken (DX), Changshun blue-eggshell chicken (CS), Lushi chicken (LS), Yunyang chicken (YY), Guifei chicken (GF), Henan gamecock (HNG), Ross 308 broiler chicken (Ross 308), Hubbard broiler chicken (HBD), Arbor Acres broiler chicken (AA), commercial H-Line brown layers (HL). Ne: effective allele numbers; Ho: observed heterozygosity; *p*-value (HWE): *p*-value of Hardy–Weinberg equilibrium. PIC: polymorphism information content. PIC > 0.50 denotes high polymorphism, 0.25 < PIC < 0.50 represents intermediate polymorphism, and PIC < 0.25 represents low polymorphism.

**Table 4:** Association of the 21-bp indel locus of *ASB9* gene with growth traits in the F_2_ resource population.

Growth Traits	Mean ± SE			*p*-Value
	II (*n* = 318)	ID (*n* = 301)	DD (*n* = 35)	
4-week weight (g)	326.43 ± 2.61	319.35 ± 2.7	310.29 ± 7.7	0.047
6-week weight (g)	574.35 ± 4.86	558.3 ± 5.03	542.44 ± 14.46	0.020
8-week weight (g)	827.83 ± 7.31	809.71 ± 7.46	783.57 ± 21.32	0.043
10-week weight (g)	1137.01 ± 9.07 ^a^	1100.62 ± 9.22 ^b^	1098.27 ± 27.37 ^ab^	0.015
12-week weight (g)	1380.52 ± 10.75 ^a^	1342.64 ± 10.96 ^b^	1286.68 ± 32.18 ^b^	0.004
12-week sternum length (cm)	9.49 ± 0.04 ^a^	9.35 ± 0.04 ^b^	9.33 ± 0.1 ^ab^	0.012
4-week shin girth (cm)	2.72 ± 0.02	2.69 ± 0.02	2.64 ± 0.04	0.011
12-week shin girth (cm)	3.88 ± 0.02 ^a^	3.83 ± 0.02 ^b^	3.81 ± 0.04 ^ab^	0.020
4-week Sternum length (cm)	6.28 ± 0.03 ^a^	6.19 ± 0.03 ^b^	6.14 ± 0.09 ^ab^	0.043
8-week Sternum length (cm)	9.0 ± 0.04	8.9 ± 0.04	8.76 ± 0.11	0.034
12-week Sternum length (cm)	11.09 ± 0.04 ^a^	10.96 ± 0.04 ^ab^	10.78 ± 0.12 ^b^	0.007
4-week body slanting length (cm)	11.46 ± 0.05	11.33 ± 0.05	11.23 ± 0.13	0.046
8-week body slanting length (cm)	16.33 ± 0.07	16.16 ± 0.07	15.9 ± 0.19	0.031
12-week body slanting length (cm)	19.9 ± 0.06 ^a^	19.69 ± 0.06 ^b^	19.71 ± 0.18 ^ab^	0.032
4-week pelvis width (cm)	5.2 ± 0.03	5.12 ± 0.03	5.04 ± 0.08	0.015

SE stands for standard error of the mean. Significant differences are shown by means with different superscripts; *p* < 0.05 is indicated by means with different lowercase letters, and *p* > 0.05 is indicated by means with the same letters.

**Table 5:** Relationship between *ASB9* gene polymorphisms and carcass traits in the reciprocal cross F_2_ population.

Traits	Mean ± SE			*p*-Value
	II (*n* = 318)	ID (*n* = 301)	DD (*n* = 35)	
SEW (g)	1120.05 ± 9.34 ^a^	1094.85 ± 9.58 ^ab^	1047.83 ± 28.2 ^b^	0.021
EW (g)	937.13 ± 8.13	912.93 ± 8.31	876.51 ± 24.48	0.018
CLW (g)	60.43 ± 0.57 ^a^	57.65 ± 0.59 ^b^	55.77 ± 1.73 ^b^	0.001
BMW (g)	72.18 ± 0.85	69.8 ± 0.87	65.92 ± 2.57	0.023
LeW (g)	152.98 ± 1.37 ^a^	148.01 ± 1.41 ^b^	140.71 ± 4.15 ^b^	0.003
LMW (g)	101.91 ± 1 ^a^	98.51 ± 1.02 ^ab^	92.98 ± 2.99 ^b^	0.004
HWP (%)	3.19 ± 0.02 ^a^	3.22 ± 0.02 ^ab^	3.35 ± 0.06 ^b^	0.020
CLR (%)	4.36 ± 0.03 ^a^	4.26 ± 0.03 ^b^	4.31 ± 0.08 ^ab^	0.023
PWP (%)	0.25 ± 0.01 ^a^	0.26 ± 0.01 ^ab^	0.28 ± 0.01 ^b^	0.009
ShW (g)	1211.34 ± 9.55 ^a^	1180.36 ± 9.82 ^ab^	1135.01 ± 29.01 ^b^	0.010
LMB (cm)	25.32 ± 0.3	24.28 ± 0.32	24.18 ± 0.91	0.047
LMD (g/cm³)	903.65 ± 17.52 ^a^	973.37 ± 18.29 ^b^	977.12 ± 53.23 ^ab^	0.018

Semi-evisceration weight (SEW), evisceration weight (EW), claw weight (CLW), breast muscle weight (BMW), leg weight (LeW), leg muscle weight (LMW), head weight percentage (HWP), claw rate (CLR), pancreas weight percentage (PWP), shedding weight (ShW), leg muscle breadth (LMB), and leg muscle density (LMD). SE stands for standard error of the mean. Significant differences are shown by means with different superscripts; *p* < 0.05 is indicated by means with different lowercase letters, and *p* > 0.05 is indicated by means with the same letters.

**Table 6:** Association of the 21-bp indel locus of *ASB9* gene with serum variable in F_2_ resource population.

Serum Variable	Mean ± SE			*p*-Value
	II (*n* = 318)	ID (*n* = 301)	DD (*n* = 35)	
Alanine aminotransferase (U/L)	1.78 ± 0.12 ^a^	1.61 ± 0.13 ^ab^	3.16 ± 0.37 ^b^	0.000

SE stands for standard error of the mean. Significant differences are shown by means with different superscripts; *p* < 0.05 is indicated by means with different lowercase letters, and *p* > 0.05 is indicated by means with the same letters.

## Data Availability

The corresponding author will provide the datasets used and analyzed during the current work on reasonable request.

## Supplementary Materials

The following supporting information can be downloaded at: https://www.mdpi.com/article/10.3390/genes14020339/s1, Table S1: *ASB9* gene proteins sequences of different species.
